# Linking Forest Planning and Recreational Trail Design: A GIS Approach for Enhancing the Social Use of Forests

**DOI:** 10.1007/s00267-025-02199-x

**Published:** 2025-06-24

**Authors:** Aitor Àvila Callau, Maitane Erdozain, Estela Inés Farías-Torbidoni, Sergio de-Miguel

**Affiliations:** 1 Forest Science and Technology Centre of Catalonia (CTFC), Solsona, Spain; 2 National Institute of Physical Education of Catalonia (INEFC), University of Lleida, Lleida, Spain; 3 Department of Agricultural and Forest Sciences and Engineering, University of Lleida, Lleida, Spain

**Keywords:** Forest management, Trail assessment, Nature-based recreation, Forest recreation, Cultural ecosystem services, Geographic Information Systems

## Abstract

Forests planned for social use are classified as such due to the cultural ecosystem services they offer. To fully benefit from these services, forest stands for social use must be easily accessible and interconnected, not just through forest roads but also through recreational trails, a key outdoor leisure infrastructure. However, forest planning and trail design are seldom integrated. This study addresses this issue by proposing a method to create connector routes between official trails and forest stands managed for social objectives (FSMSO), enhancing the socio-recreational use of forests. Using Geographic Information Systems (GIS), our approach analyses overlap between official trails and FSMSO, identifies direct routes with origin-destination matrices and assesses FSMSO accessibility. Route viability was then calculated, supporting decision-makers in assessing route homologation potential. In our study area (Catalonia, Spain), findings show that only 14% of the FSMSO overlap with official trails. Among those not overlapping, 75% are connected with official trails via the road network, while 25% are inaccessible. Of the accessible stands from official trails, 54% are more than 20 min away on foot, while 22% are within 20 min. Most created connectors (62%) have moderate viability, with 13% showing high viability for official homologation. Regarding forest types, riparian forests are the most common in FSMSO (15%) and the most connected to official trails (17%). Our methodology supports integrated forest planning and trail design, enhancing socio-recreational opportunities, while emphasising the need for regulations addressing risks and challenges linked to promoting the public use of forests.

## Introduction

Forests provide multiple ecosystem services (ES) to societies, including provisioning (timber, food, freshwater), regulating (climate and water regulation, carbon sequestration, erosion prevention, pollination), cultural (spiritual, recreational, aesthetic) and supporting (primary production, soil formation, nutrient and water cycle) (Caglayan et al. 2020; Lupp et al. 2022; MEA Millennium Ecosystem Assessment 2005). Cultural ES (CES), associated with the social use of forests (Krsnik et al. 2023), are becoming increasingly crucial over traditional forest uses (Füger et al. 2021; Kaplan 1995) because spending time in forests improves human physical and mental health (Calogiuri and Elliott 2017; Dudek 2017; Turgut et al. 2020). Consequently, outdoor recreation is a significant forest CES, with people seeking nature to escape urban stress (Agimass et al. 2018; Caglayan et al. 2020; Dağıstanlı et al. 2018). As a result, designing outdoor recreation infrastructures to facilitate access to forests is becoming a priority on political agendas worldwide (Lingua et al. 2022, 2023). Trail-related activities are a vital part of recreation infrastructure (Snyder et al. 2008). Recreational trails for walking, cycling and running promote exercise and active transportation, enhancing users’ health, quality of life and well-being (Àvila Callau et al. 2023; Brownson et al. 2000; Corning et al. 2012; Wiggs et al. 2008) while mitigating urban health issues like lack of exercise and mental health problems (Barton and Pretty 2013; Lingua et al. 2022).

In recent decades, forest planning has increasingly embraced the paradigm of multifunctional management, which integrates not only productive functions but also socio-cultural and ecological values (Caglayan et al. 2021; Meo et al. 2015; Vadell et al. 2022). Within this framework, CES (such as aesthetic enjoyment, spiritual enrichment, recreational use, and educational value) have gained particular attention (Lingua et al. 2022, 2023; Termansen et al. 2013; Tudoran et al. 2022). While some scholars use CES and multifunctional forest management interchangeably, others distinguish between them conceptually. In this study, we adopt the view that CES constitute a core component of multifunctional forest management, which seeks to balance the delivery of ecosystem services across both productive and non-productive domains (Meo et al. 2015; Torralba et al. 2020).

Despite their importance, the integration of recreational values into forest management remains challenging due to methodological difficulties in identifying, mapping, and quantifying these services (Lingua et al. 2022, 2023; Termansen et al. 2013; Tudoran et al. 2022). The factors influencing the recreational value and demand of forests include natural area characteristics, availability of facilities, user demographics and user preferences (Agimass et al. 2018; Giergiczny et al. 2015). Additionally, accessibility is crucial, with citizens preferring forests near roads (Agimass et al. 2018; Neuvonen et al. 2007; Tudoran et al. 2022) and forests near urban or peri-urban areas with extensive networks of paths or trails (Àvila Callau et al. 2023; Meo et al. 2015; Tudoran et al. 2022).

Route planning and recreational trail design increase interest in natural areas and promote outdoor physical activity by providing access to these spaces (Turgut et al. 2020). Evaluating trail infrastructure suitability for hiking in natural areas is common (e.g., Vías et al. 2014, 2018; Vías and Ocaña 2014), but trail design is time-consuming and considers various factors for optimal location and design (Kokkinidis et al. 2013). Factors such as topography, landscape types, recreational infrastructure and attractions are considered when determining suitable hiking routes (Turgut et al. 2020). Additionally, hikers prefer trails that offer interest, challenge, experiences and safety (Snyder et al. 2008). The environmental impacts of trails are also a concern, but proper trail design can reduce these risks (Snyder et al. 2008). Furthermore, usability depends on trail connectivity to access points and other trails, requiring careful consideration of start and end points and existing trails (Kokkinidis et al. 2013).

While recreational trail design plays a crucial role in increasing interest and physical activity in natural areas, the integration of forest roads further supports these efforts by enhancing multifunctionality and accessibility. Forest roads support forestry, operational activities and social functions like recreation (Laschi et al. 2016; Picchio et al. 2019). However, evaluating forest roads according to the social function of forests to design trails enhances their multifunctionality and improves forest recreation (Hruza and Vyskot 2010). In this context, Geographic Information Systems (GIS) enhances trail design efficiency by utilising configurable data and algorithms like the least-cost path to automate the generation and evaluation of optimal routes in recreational trail planning (Kokkinidis et al. 2013; Snyder et al. 2008).

The scientific literature has not addressed the connection between official trails and forest stands managed for social objectives (FSMSO). Considering the growing societal demand for recreation opportunities in forests and the complexity behind trail design, this article proposes a method to create connecting routes that optimise forest socio-recreational use and evaluate FSMSO accessibility. The study aimed to (1) analyse the spatial overlap between official trails and FSMSO; (2) identify the shortest routes connecting them; (3) measure the accessibility of FSMSO using these routes; and (4) assess the viability of these routes as official connectors. Homologating forest trails ensures they are used optimally for social and recreational purposes (Hruza and Vyskot 2010). Based on this, the last objective supports decision-makers and trail designers in evaluating routes to be proposed as official connecting axes between existing official trails and FSMSO. GIS methods were used to create routes based on origin-destination matrices. While the region of Catalonia (in northeastern Spain) was used as a case study, the method has global applications.

## Methods

**Fig. 1:**
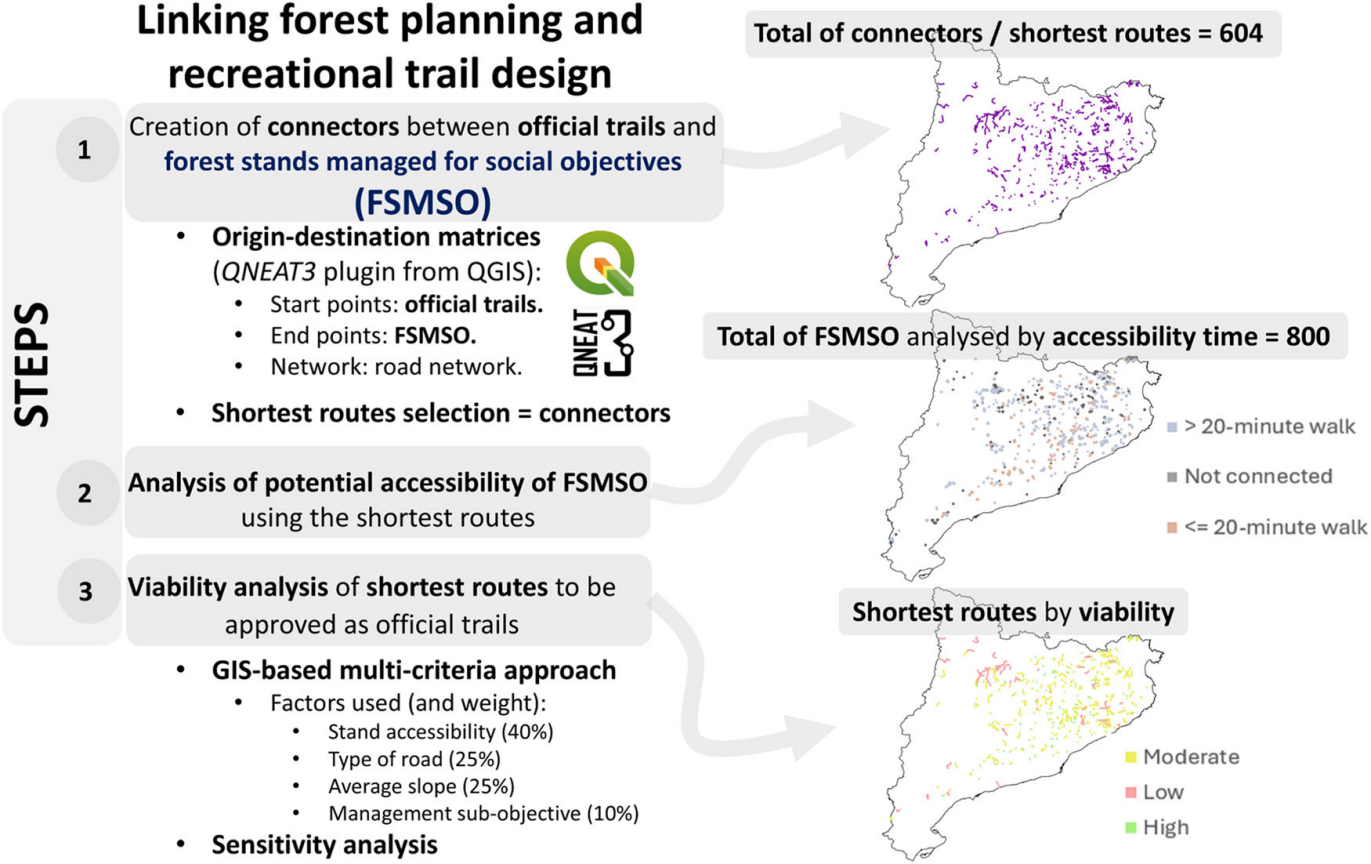
Diagrammatic overview of the study’s methodological framework

This study is divided into three main steps (Fig. 1). First, connectors are created between official trails and FSMSO using origin-destination matrices generated with the QNEAT3 plugin in QGIS, resulting in 604 shortest routes. Second, the accessibility of 800 FSMSO is analysed by calculating walking time along the shortest routes, classifying them into categories. Third, a viability analysis of the routes is conducted using a GIS-based multi-criteria approach. This analysis prioritises the routes according to four weighted factors: stand accessibility, type of road, average slope, and forest management sub-objective.

### Study Area, Data Sources and Collection

**Fig. 2:**
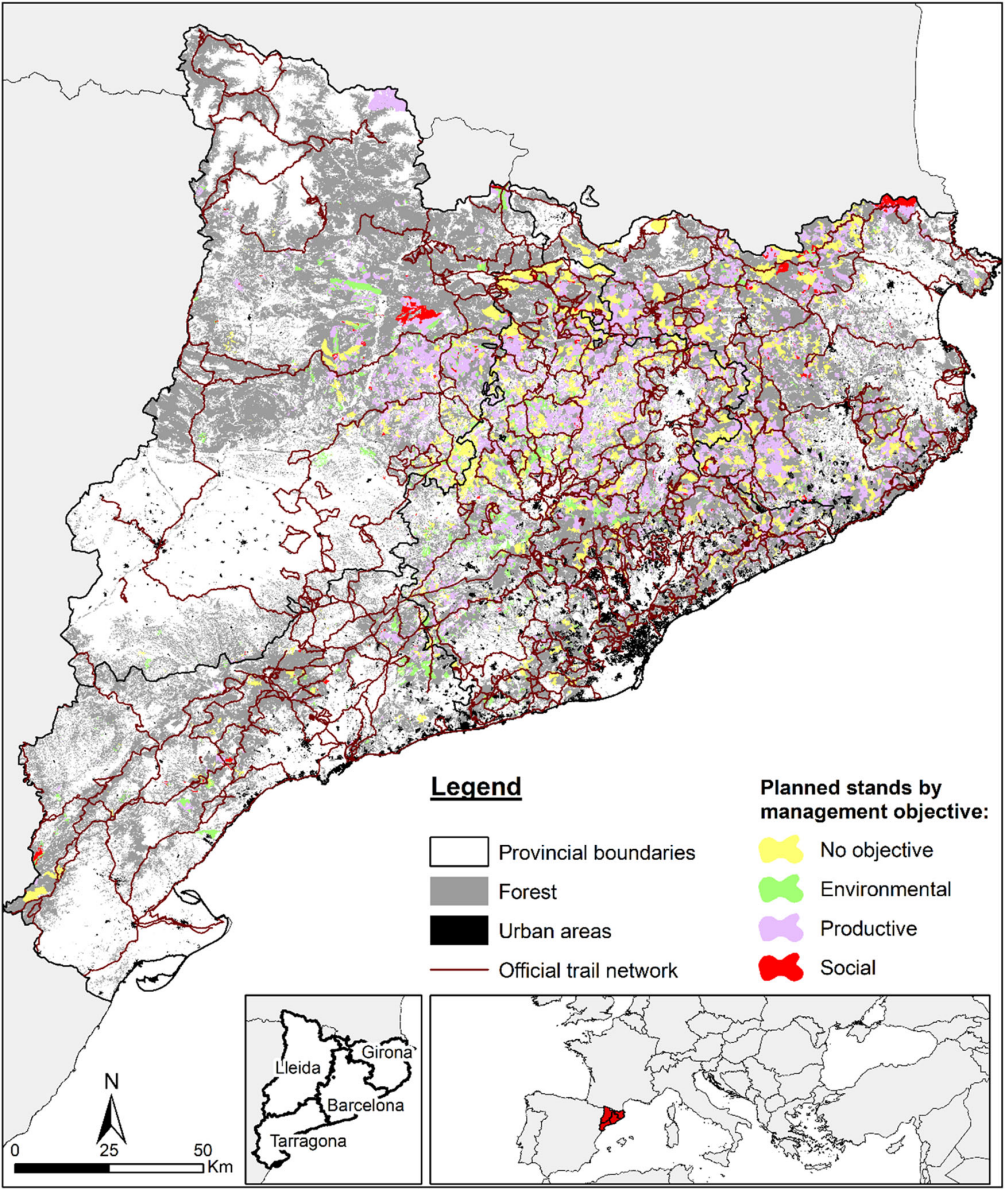
Study area with official trails and planned stands by management objective. Note: The administrative boundaries are provided by the Cartographic and Geological Institute of Catalonia (ICGC), while the forest and urban layers are sourced from the Spanish Forest Map at 1:25,000 Scale (MFE25)

The study area was the Catalonia region in northeast Spain (Fig. 2), covering approximately 32,000 km², with 64% of the area being forested (Statistical Institute of Catalonia 2022). According to the Forest Ownership Centre (2024), 76% of the forest area in Catalonia is privately owned, while the remaining 24% is public. Approximately 67% of the forest area is not covered by a forest management plan, whereas 33% has undergone some form of planning. Of the managed forest area, 73% is privately owned and 27% public. Notably, forest planning in private forests has grown significantly over the past two decades, expanding from around 100,000 hectares in the late 1990s to more than 450,000 hectares today. On the other hand, public funding limitations together with the fact that a considerable proportion of the total forest area corresponds to non-wooded rangelands or to woodlands with low economic profitability explains the limited percentage of forest area under forest planning compared to the total forest area. According to the regulation on forest management planning within the study region, the planning horizon of new forest management plans may vary between 10 and 30 years (Forest Ownership Centre 2011).

Based on our GIS-based calculations, Catalonia has a significant network of official hiking trails, exceeding 10,000 km and accounting for around 20% of Spain’s trail network (Fig. 2). We combined official trail shapefiles obtained from the Spanish National Geographic Institute (IGN) and homologised by three different institutions: (1) the trails for walkers of Long Distance (GR), Short Distance (PR) and Local Trails (SL) approved by the Spanish Federation of Mountain Sports and Climbing (FEDME); (2) the “Natural Paths” of the Natural Paths Program of the Spanish Ministry of Agriculture, Fisheries and Food; and (3) the “Green Routes” of the Spanish Railways Foundation, i.e., converted old railway routes for walkers and cyclists. These data sources were chosen for their completeness, standardisation, institutional validation, and their public accessibility and downloadability within the geographical context of the study area.

The information on the spatial distribution of planned forest stands (stands under a multifunctional forest management plan) and their forest management objectives (Fig. 2) was retrieved from the non-public spatial database on forest planning managed by the Forest Ownership Centre from the Government of Catalonia. In this database, only stands with an approved forest management plan are categorised into four planning objective categories (productive, environmental, social, and undetermined), with further classification into sub-objectives. The “undetermined” category refers to stands for which no information on objectives is available in the database, the reason for this absence remaining unreported.

These objectives are determined based on the guidelines provided in the manual for drafting the aforementioned management plans (Forest Ownership Centre 2011), which must be followed by the technical teams responsible for their preparation. The manual assumes that forests are inherently multifunctional but, within this framework, it allows the identification of primary or preferential objectives for the area under planning, classified into productive, environmental, and social categories. Further specifications for determining these objectives are detailed in the manual. In the case of social objectives, the manual specifies that certain singular landscape or cultural elements may justify specific management interventions focused on their preservation, maintenance, and/or improvement, beyond traditional or more production-oriented management approaches. These social objectives are further detailed through sub-objectives, which encompass management approaches aimed at promoting (1) the valorisation of the landscape and/or natural and unique cultural elements; (2) the preservation of forested areas or spaces; (3) the facilitation of recreational and leisure uses of the forest environment; and (4) other similar purposes. The objectives, sub-objectives and the forest type (i.e., dominant tree species) were used as thematic information in this work.

### Overlap Analysis and Creation of Connectors between Official Trails and Forest Stands Managed for Social Objectives

**Fig. 3:**
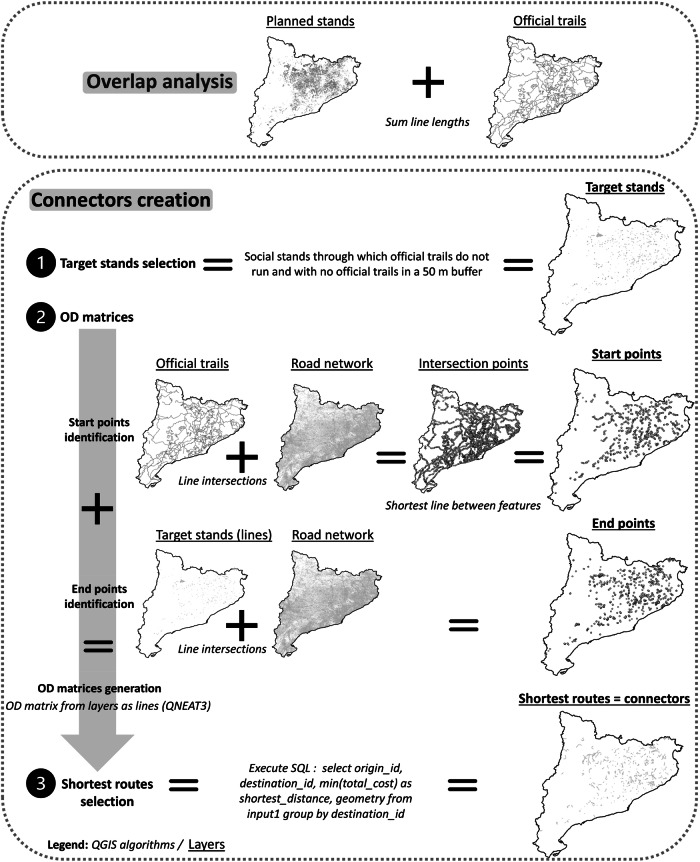
Methodological structure of the overlap analysis and the creation of connectors between official trails and forest stands managed for social objectives

We first analysed the overlap between the forest stands and official trails (Fig. 3). Using the “sum line lengths” algorithm in QGIS 3.3, we determined which planned stands were connected to the official trail network and calculated the accumulated length of trails within each stand.

Next, we identified the shortest routes connecting the official trails to FSMSO, i.e., the forest stands with the highest potential for CES and outdoor recreation (Fig. 3). To select the target FSMSO, we focused on stands that (1) have a social management objective, (2) are not crossed by official trails and (3) have no official trails within a 50-m buffer. The latter were excluded due to their proximity to the trails.

Once the stands were selected, we generated origin-destination matrices to determine the potential routes between the official trails and the target FSMSO to select and extract the shortest route. We used origin-destination matrices because new recreation access models should rely on network distances, not Euclidean ones (Kearns and Deng 2019). We used the “OD matrix from layers as lines” module in the QNEAT3 (QGIS Network Analysis Toolbox) plugin (QGIS 3.3) to calculate these matrices, which map routes based on a network layer, starting points and endpoints.

Firstly, to select starting points on the official trails, we overlapped the road network with the trails using QGIS’s “line intersections” algorithm because we considered that the road network allows connections between a given trail and a given forest stand. The road network is derived from the “transport networks” layer downloaded in shapefile format from the IGN. However, the official trail network was generated from traces recorded with Global Navigation Satellite System (GNSS) devices, which do not align perfectly with the road network layer. This misalignment created excessive vertices where the two layers intersected, leading to inaccuracies. To accurately match the trails to the road network and reduce the number of vertices in the starting points layer, we used the “snap geometries to layer” algorithm in QGIS, which aligned the trail layer with the road network layer and allowed us to extract the actual intersections (starting points). We then used QGIS’s “shortest line between features” to identify the ten closest starting points for each endpoint in the target stands, which reduced the number of starting points and iterations needed for the origin-destination matrices. We created sets for each stand, pairing each endpoint with its ten nearest starting points, resulting in multiple origin-destination matrices.

Secondly, to calculate the destination points for the target forest stands, we extracted the vertices where the stand perimeters intersected the road network. We converted the stand layer from polygons to lines and then intersected these lines with the road network using the “line intersections” algorithm, generating the destination points.

Finally, after preparing the starting points (from the trail) and destination points (in the FSMSO), we used the “OD matrix from layers as lines” to find the shortest routes between each starting point and endpoint. The road network layer served as the network layer, excluding roads prohibited for pedestrians. We used “shortest” as the optimisation criterion and set the “matrix geometry follows routes” parameter to ensure the routes followed the road network instead of straight lines. The attribute table of the resulting layer contained the identifier of the origin points (“origin_id”), the identifier of the stand to which the destination points belonged (“destination_id”) and the total cost (or total distance) in meters (“total_cost”). An SQL query (Fig. 3) was performed to find the shortest route from the official trails to each target stand.

After identifying the shortest routes between official trails and FSMSO, we added road network attributes (e.g., road type, ownership) and calculated slope parameters using QGIS 3.3’s “calculate trail elevation statistics” algorithm with a digital elevation model of the study area.

### Analysis of Potential Accessibility of Forest Stands for Social Use

We calculated the potential accessibility (in time) of the FSMSO considering the hypothetical case of a hiker who decided to deviate from the official trail to reach a given forest stand using the length of the route (“total_cost” field) and assuming an average hiking speed of 5 km/h based on previous studies (Bohannon 1997; Stoia et al. 2022). Following Stoia et al. (2022), the analysis was conducted using time intervals of 5, 10, 15, 20 and more than 20 min to classify forest stand accessibility.

### Viability Analysis of Shortest Routes that Connect Official Trails with Forest Stands for Social Use

The identified routes connecting the official trail network with the FSMSO varied in characteristics, affecting their suitability as connecting axes. We calculated a viability index for each route based on its characteristics, providing a prioritisation criterion for managers. The viability index was developed using a GIS-based multi-criteria approach, incorporating four key factors: accessibility, road type, average slope, and forest management sub-objectives. These are the factors we identified as most directly related to the technical and operational feasibility of the routes from a management perspective. By focusing on these variables, our approach provides a systematic and objective evaluation, offering clear criteria for route prioritisation by managers.

Furthermore, we subdivided the four factors into categories and assigned a score from 0 (lowest viability) to 1 (highest viability) (Table 1). The following statement justifies the assignment of the scores: the greater the accessibility, the more suitable the type of road, the smaller the slope and the more in line the sub-objective of the stand with outdoor recreation, the greater the final viability of the route. In summary, this viability index provides a systematic way to prioritise routes, assisting managers in evaluating and selecting the most viable routes for official trail connections.

**Table 1:** Factors, categories and scores assigned in the viability analysis

Factors (assigned weight)	Categories	Score (0-1)
Stand accessibility (40%)	≥5 min	1
	(5–10] min	0.75
	(10–15] min	0.5
	(15–20] min	0.25
	>20 min	0
Type of road through which the route runs (25%)	>75% by paths and trails	1
	50–75% by paths and trails	0.5
	≤50% by paths and trails	0
Average slope of the route (25%)	≤3%	1
	(3–10] %	0.75
	(10–20] %	0.5
	(20–30] %	0.25
	>30%	0
Management sub-objective of the stand connected by the route (10%)	Recreational uses of the forest environment	1
	Valorisation of the landscape and/or natural and unique cultural elements	0.66
	Other	0.33
	Preservation of forest spaces	0

The type of road was included following the Qualitätsweg Wanderbares (Quality Hiking Trail) Guide (Deutscher Wanderverband Service 2022) promoted by the German Hiking Association (DWV), which favours routes with a higher percentage of trails and dirt tracks, as paved roads reduce naturalness and environmental integration. Slope categories were based on previous studies on forest recreational potential (e.g., Caglayan et al. 2020; Vías and Ocaña 2014).

After assigning scores to each route for the four factors, we weighed the factors, prioritising accessibility as the most important (Table 1), given that better accessibility increases the likelihood of hikers choosing a route off the official trail (Ciesielski and Stereńczak 2018). A weighted linear sum was calculated to obtain the viability index. Finally, we categorised each route based on its viability index using three intervals: low (0–≤0.33), medium (0.33–≤0.66) and high (0.66–≤1).

To assess the robustness of our viability index, we conducted a sensitivity analysis inspired by Caglayan et al. (2020). Eight scenarios were considered, half of which involved increasing the weight of a factor by 10%, while the other half decreased it by 10%. In each scenario, adjustments were made to the weights of the remaining factors proportionally to maintain a total weight of 100%.

## Results

### Spatial Overlap between Official Recreational Trails and Forest Stands Managed for Social Objectives

Of the 36,342 planned stands in Catalonia (Table 2), more than half (55.77%) had a productive objective, 9.75% had an environmental objective and 2.75% had a social objective. Around a third of the planned stands (31.73%) had no specific objective assigned. Accumulated area results mirrored those reported for number of stands (Table 2), as the average stand size is approximately 10 ha. Only 11.75% of the planned stands contained official trails within their perimeter. Among these, stands with a social objective had the highest proportion at 13.83%. The remaining categories exhibited similar proportions, each around 10%.

**Table 2:** Characteristics of forest stands by management objective

	Planed forest stands				Planed forest stands through which official trails run	
	*n*	%	Accumulated area (ha)	Accumulated area (%)	*n*	%
Productive	20,269	55.77	244,957.73	53.28	2422	11.95
Environmental	3542	9.75	51,805.47	11.27	456	12.87
Social	998	2.75	14,616.68	3.18	138	13.83
Undetermined	11,533	31.73	148,413.34	32.28	1255	10.88
Total	36,342	100.00	459,793.22	100.00	4271	11.75

Regarding management sub-objectives (Table 3), 40.78% of the FSMSO focused on preserving forest spaces, 27.05% on enhancing landscape and cultural elements, 23.05% on recreational use and 9.12% were labelled Other. Among the FSMSO crossed by official trails, 44.20% aimed at preserving forest spaces, while 31.88% were dedicated to landscape and cultural elements, and 15.22% were intended for recreational use. *Quercus ilex* forests were the most common type in these areas (15.23%), followed by riparian forests, plantations and *Pinus halepensis* forests. *Quercus ilex* forests also had the highest overlap with trails (21.02%), with riparian forests and the *Pinus* forests also showing significant trail overlap.

**Table 3:** Characteristics of forest stands managed for social objectives by management sub-objective and forest type

		FSMSO		FSMSO through which official trails run			Length of official trails that run through FSMSO	
		*n*	%	*n*	%	Area: mean (ha)	Sum (Km)	Mean (Km)
Sub-objective	Preservation of forest spaces	407	40.78	61	44.20	22.80	19.61	0.32
	Valorisation of the landscape and/or natural and unique cultural elements	270	27.05	44	31.88	16.30	12.78	0.29
	Recreational uses of the forest environment	230	23.05	21	15.22	17.76	7.73	0.37
	Other	91	9.12	12	8.70	5.94	2.56	0.21
	Total	998	100	138	100	-	42.67	-
Forest type	*Quercus ilex* forest	152	15.23	29	21.02	27.95	12.92	0.44
	Riparian forests	149	14.93	14	10.14	6.21	1.30	0.09
	Plantations	77	7.72	4	2.90	0.91	0.59	0.15
	*Pinus halepensis* forests	61	6.11	12	8.70	7.66	1.49	0.12
	*Pinus sylvestris* forest	40	4.01	7	5.07	26.50	2.87	0.41
	Mixed forests of *Quercus ilex* and *Pinus halepensis*	33	3.31	2	1.45	17.34	2.36	1.18
	Other forest types	486	48.70	70	50.72	-		-
	Total	998	100	138	100	-	42.67	-

Only forest types found in more than 30 forest stands (percentile 90) are represented. The “plantations” category includes different species (pine, black poplar, cedar, etc.)

### “Official trail – FSMSO” Connectors

**Fig. 4:**
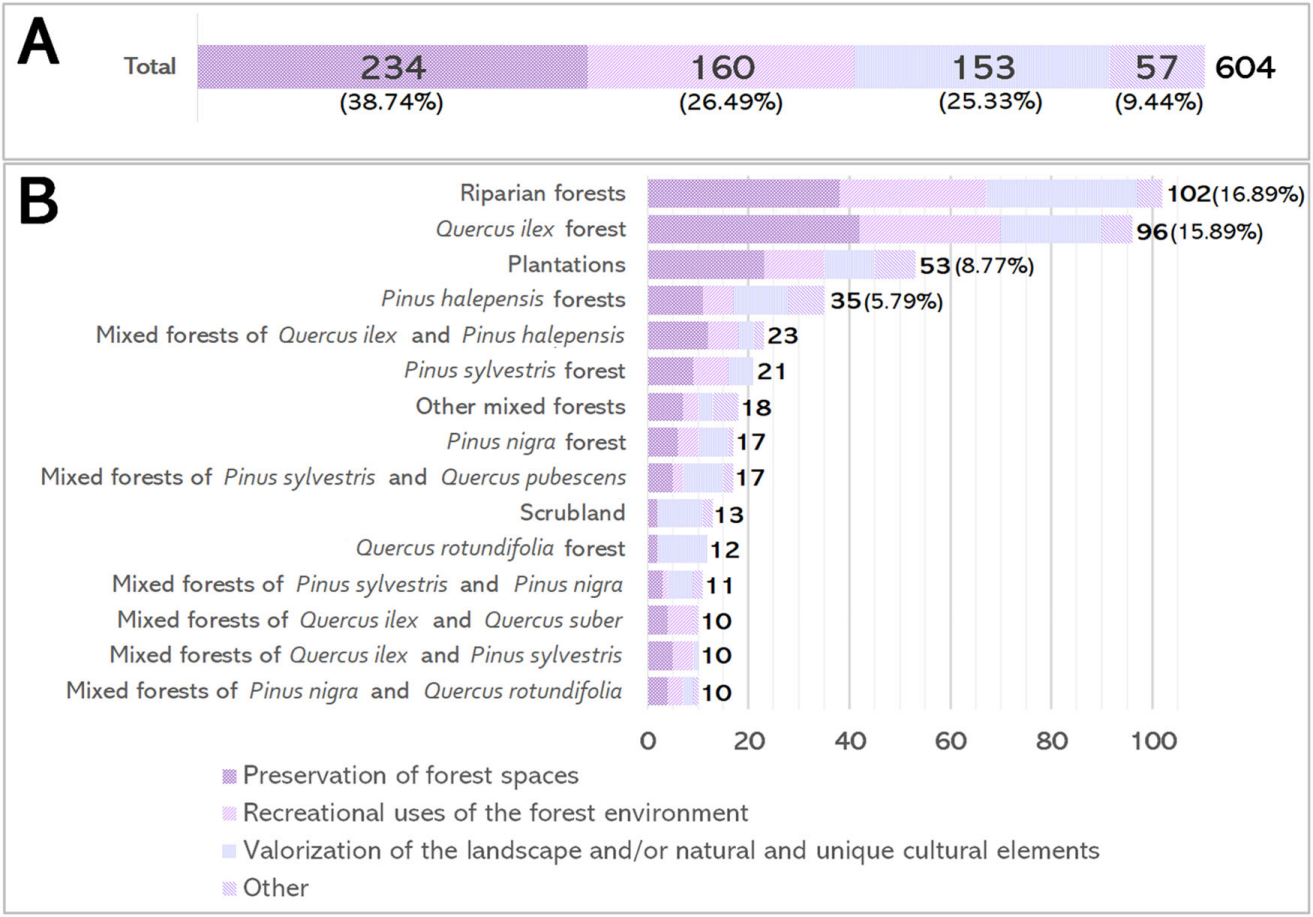
Shortest routes between official trails and forest stands managed for social objectives by **A** the management sub-objective of the connected stand and by **B** the forest type and the management sub-objective. Note: Only forest types in ten or more stands appear on the graph. Additionally, only percentages greater than 5% are shown

Out of 800 forest stands analysed, 75.50% were accessible via road network. In the remaining 24.50%, no connection between the FSMSO and the official trails could be made through the road network. Consequently, 604 routes were established to connect these stands to the official trail network (Fig. 4). These routes totalled 3130 km in length, averaging 4.5 km in distance and taking an average of 54.37 minutes to walk. Among all routes generated, 38.74% connected stands focused on Preservation of forest spaces (Fig. 4A), followed by stands dedicated to Recreational uses of the forest environment (26.49%) and Valorisation of the landscape and/or natural and unique cultural elements (25.33%). Regarding forest types connected (Fig. 4B), riparian forests had the highest proportion of connections (16.89%), followed by *Quercus ilex* forests, plantations and *Pinus halepensis* forests. Together, these four forest types represented over 40% of all connected stands, while other types had smaller proportions (less than 5%).

**Fig. 5:**
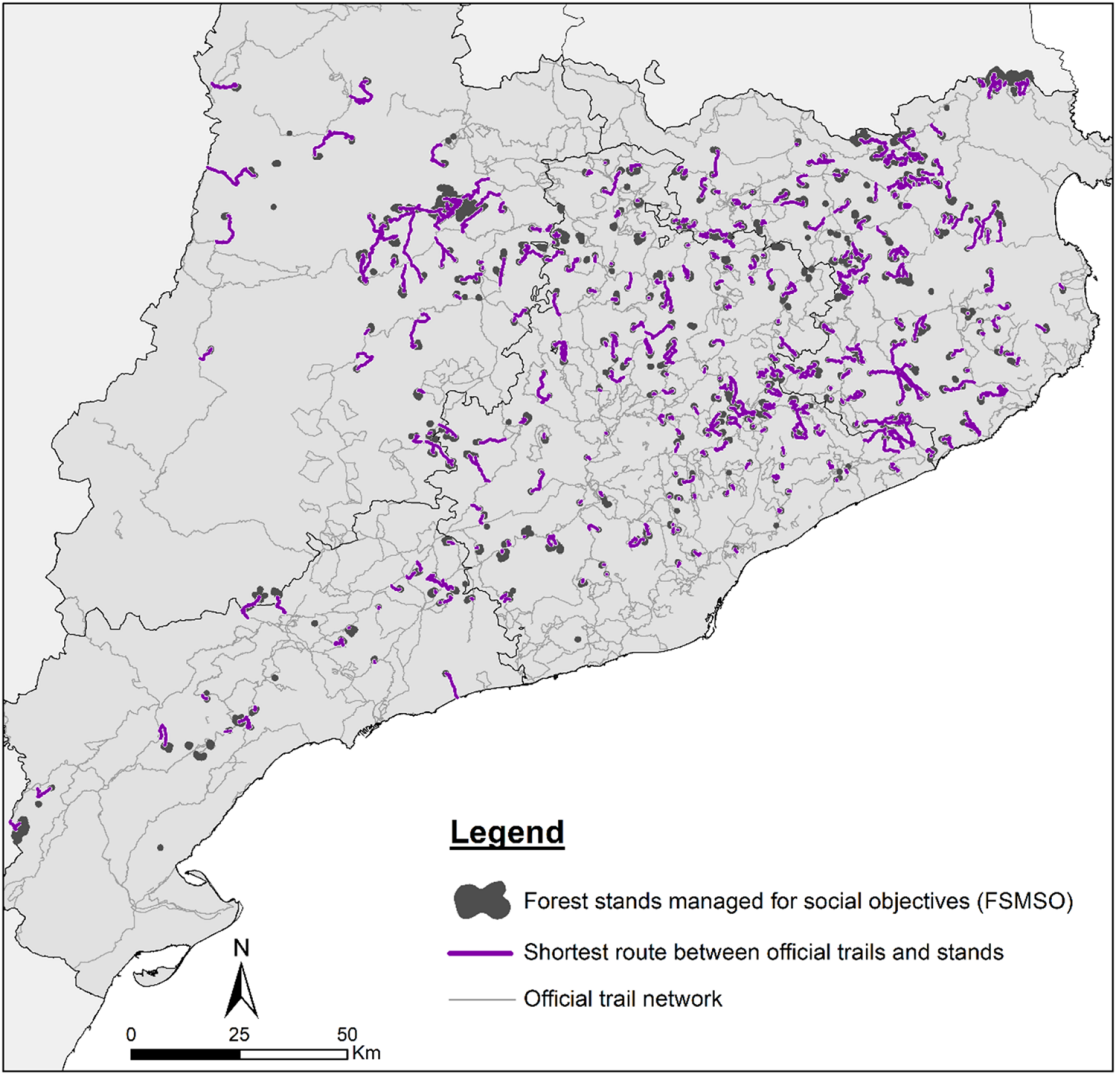
Shortest routes between official trails and forest stands managed for social objectives. Note: The size of the stands and routes is enlarged to improve their visualisation

The shortest routes between official trails and FSMSO are distributed across different sectors (Fig. 5). Longer routes are mainly found in the northwestern sector of the study area, indicating that the FSMSO are farther from the official trail network there. In contrast, stands in the central sector are closer to the official trails, resulting in shorter routes.

Among the 3130 km of routes connecting stands to official trails (Table 4), paths comprised the majority (60.26%), followed by sections of conventional roads (28.82%) and trails (8.41%). Urban sections or multilane roads made up only a tiny fraction. Ownership details showed that the majority (74.16%) of route lengths had unknown ownership, while the Catalan Government owned 16.60% and 6% by county councils. Regarding slope, a significant portion (82.89%) of the routes had an average slope between 3% and 10%. Routes between 10% and 20% slope accounted for 10.17%, while those with a flatter terrain (≤3%) represented 6.7%.

**Table 4:** Characteristics of shortest routes by road type and road ownership

		Length (km)	Length (%)
Road type	Conventional road	901.94	28.82
	Urban road	77.58	2.48
	Path	1,886.19	60.26
	Trail	263.30	8.41
	Multilane road	1.00	0.03
Road ownership	General State Administration	22.09	0.71
	City hall	79.62	2.54
	Unknown	2,321.09	74.16
	Autonomous Community	519.49	16.60
	County council	187.71	6.00
Average slope	≤3%	209.86	6.70
	(3–10] %	2,594.51	82.89
	(10–20] %	318.26	10.17
	(20–30] %	7.18	0.23
	>30%	0.19	0.01

The term “path” in official road categories typically denotes a rural or forest road suitable for vehicle traffic. On the other hand, “trail” refers to a narrower pathway primarily intended for pedestrians or cyclists

### Potential Accessibility of Forest Stands Managed for Social Objectives

**Fig. 6:**
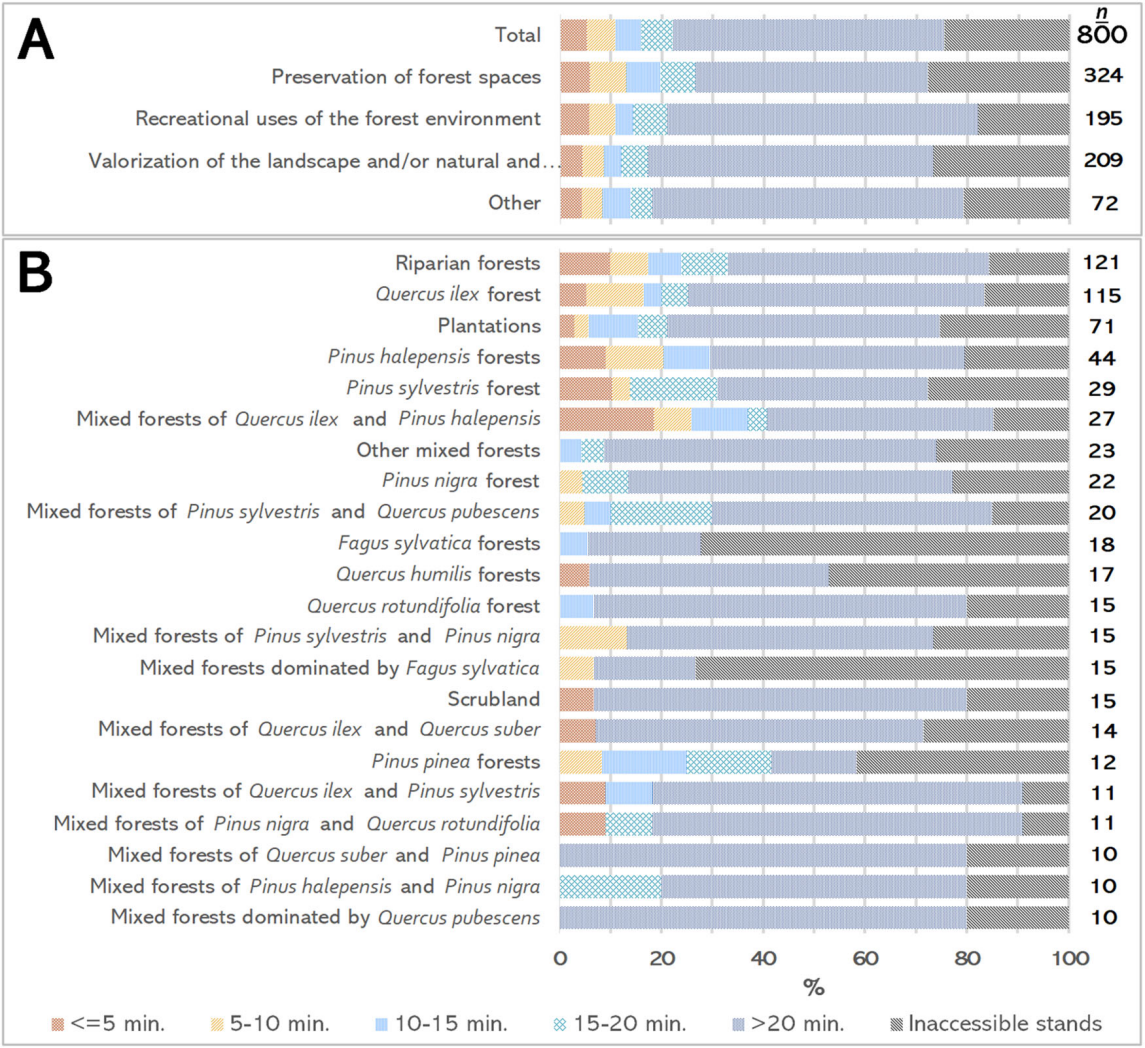
Percentage of forest stands managed for social objectives by **A** management sub-objective (rows) and accessibility time (colours) and by **B** forest type (rows) and accessibility time (colours). Note: Only forest types found in ≥10 stands are shown

Of all the FSMSO analysed (Fig. 6A), over half (53.50%) were situated more than a 20-min walk from the nearest official trail. Another 24.50% could not connect to any official trails, while the remaining 22% were within a 20-min walk. Trends were similar for different sub-objectives (Fig. 6A). In terms of forest type (Fig. 6B), the most accessible (within 5 min walking distance) were mixed forests of *Quercus ilex* and *Pinus halepensis* (18.52%), *Pinus sylvestris* forests (10.34%) and riparian forests (9.92%). *Pinus pinea* forests were highly accessible (up to 20 min walking, 41.67%), followed by mixed forests of *Quercus ilex* and *Pinus halepensis* (40.74%). The least accessible (>20 min) included mixed *Quercus suber* and *Pinus pinea* forests (80%), *Quercus pubescens* dominated forests (80%), scrubland (73.33%) and *Quercus rotundifolia* stands (73.33%), among others. *Fagus sylvatica* forests, both pure and mixed, remained largely inaccessible (about 73%). *Quercus humilis* forests (47.06%) and *Pinus pinea* forests (41.67%) also had relevant percentages of inaccessibility.

**Fig. 7:**
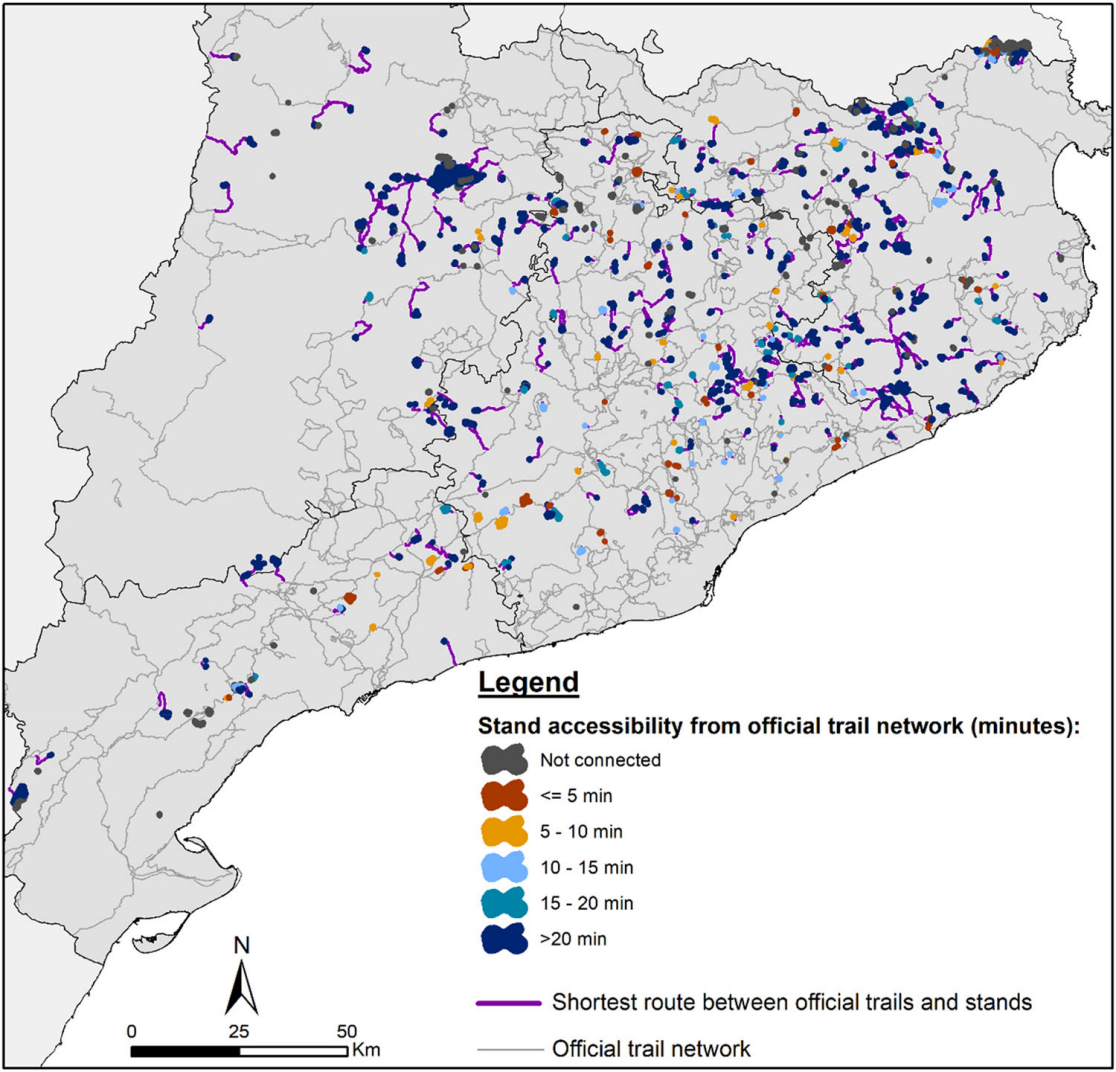
Forest stands managed for social objectives by accessibility time. Note: The size of the stands and routes is enlarged to improve their visualisation

Patterns of accessibility from official trails (Fig. 7) were not equally distributed across the territory. The most accessible stands were concentrated in regions with an extensive trail network, notably in much of the central area of the study region. Conversely, areas with fewer official trails, such as the northwestern and northeastern sectors, had stands requiring more than 20 min to reach. Examples include the northwestern and northeastern parts of the study region. Stands that could not connect to the official trail network were scattered spatially.

### Viability Proposal for the Design of Trail Connectors between Official Trails and Forest Stands Managed for Social Objectives

**Fig. 8:**
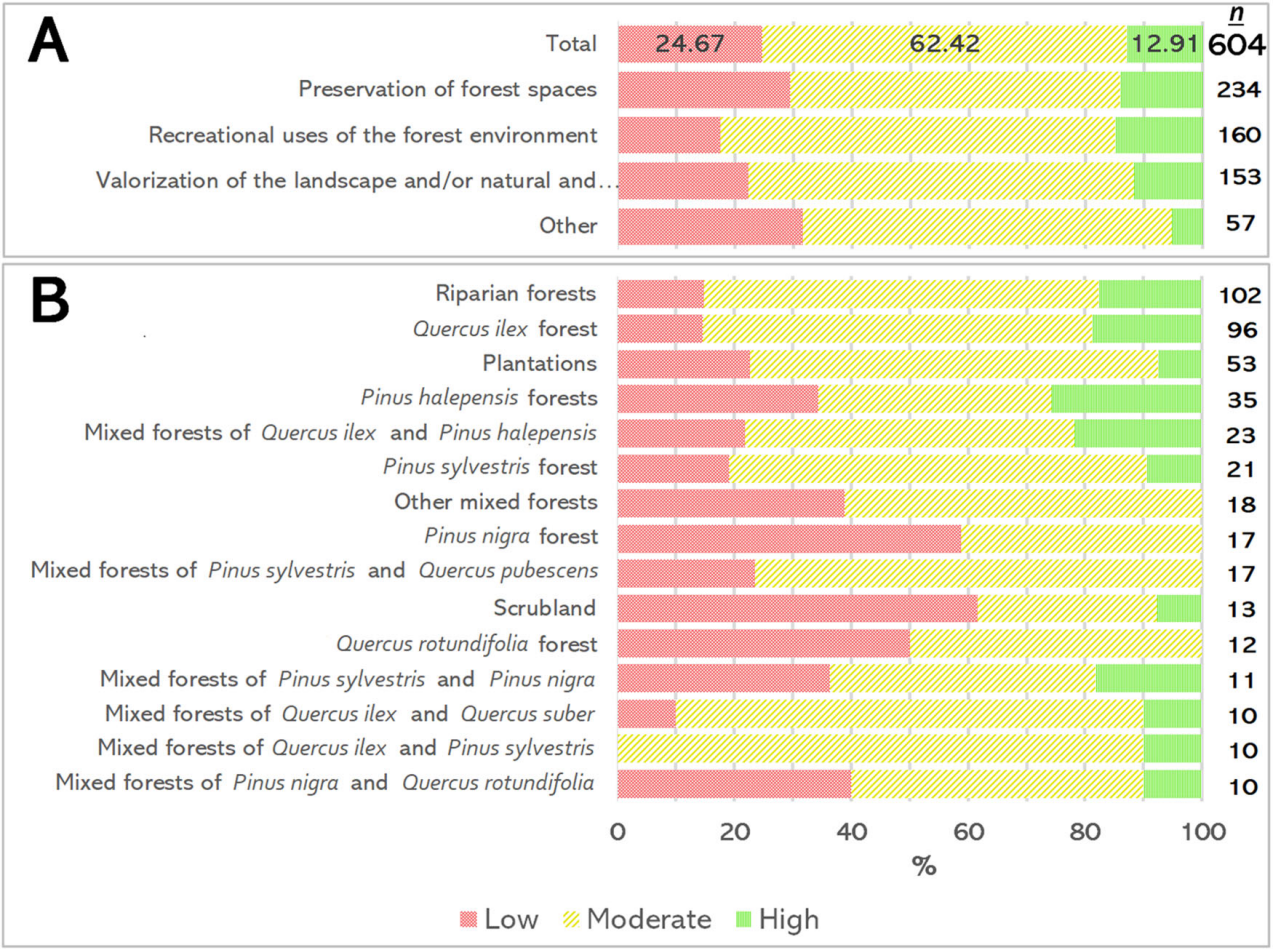
**A** Percentage of connected forest stands managed for social objectives by management sub-objective and viability of the route that connects them and **B** by forest type and viability. Note: Only forest types found in ≤10 stands are shown

Most proposed connectors (62.42%) between official trails and FSMSO were considered moderately viable, while 25.67% had low viability and only 12.91% had high viability to be proposed as official connecting axes. These trends were comparable across stands with different sub-objectives (Fig. 8A).

Regarding forest type (Fig. 8B), stands with high viability routes included *Pinus halepensis* forests (25.71%), mixed *Quercus ilex* and *Pinus halepensis* forests (21.74%), *Quercus ilex* forests (18.75%), mixed forests of *Pinus sylvestris* and *Pinus nigra* (18.18%) and riparian forests (17.65%). In contrast, scrubland (61.54%), *Pinus nigra* forests (58.82%) and *Quercus rotundifolia* stands (50%) had higher proportions of routes with low viability.

**Fig. 9:**
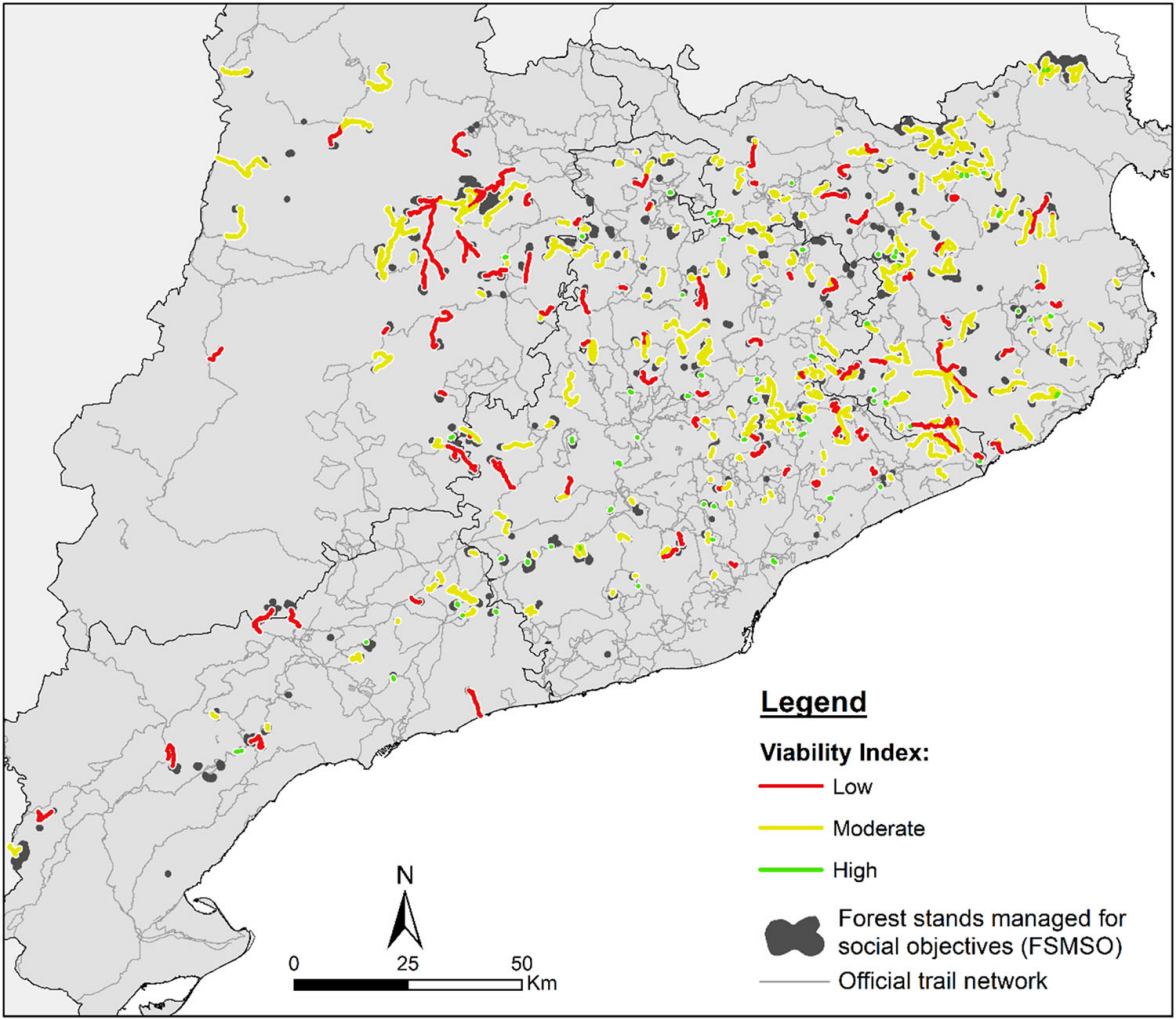
Shortest routes by viability level. Note: The size of the stands and routes is enlarged to improve their visualisation

Routes with high viability were mostly concentrated in the central area of the study region (Fig. 9). In contrast, the least viable routes were predominantly found in the northwestern area, with some also in the northeastern area. Routes with medium viability showed a more evenly distributed spatial pattern.

The sensitivity analysis indicated that in most scenarios (Table 5), around 10% of stands changed viability categories compared to the original solution. The most notable changes occurred when decreasing the weight of the accessibility factor and increasing the weights of the management sub-objective and road type factors (all by 10%). Importantly, no shifts from low to high or high to low viability were observed in the sensitivity analysis.

**Table 5:** Number of stands that change their category in the different proposed scenarios

Scenarios		L to M	L to H	M to L	M to H	H to L	H to M	Total change	Total change (%)
Accessibility	Increased 10%	0	0	50	6	0	2	58	**9.60**
	Decreased 10%	61	0	0	11	0	9	81	**13.41**
Road type: path	Increased 10%	28	0	14	24	0	2	68	**11.26**
	Decreased 10%	32	0	12	6	0	9	59	**9.77**
Average slope	Increased 10%	45	0	0	15	0	2	62	**10.26**
	Decreased 10%	0	0	26	4	0	1	31	**5.13**
Management sub-objective	Increased 10%	33	0	13	9	0	19	74	**12.25**
	Decreased 10%	28	0	16	15	0	3	62	**10.26**

*L* low, *M* Moderate, *H* High

## Discussion

### Strengths, Applicability and Limitations

The methodology presented is a valuable tool for identifying and evaluating connector routes to expand official trail networks and connect them to forests with recognised social functions. This approach supports effective management planning, enhancing the social and recreational use of these ecosystems. Its scalability allows for adaptation to diverse objectives by prioritising different factors in route viability assessments, enabling decision-makers to propose viable connector routes for inclusion in official trail networks.

**Fig. 10:**
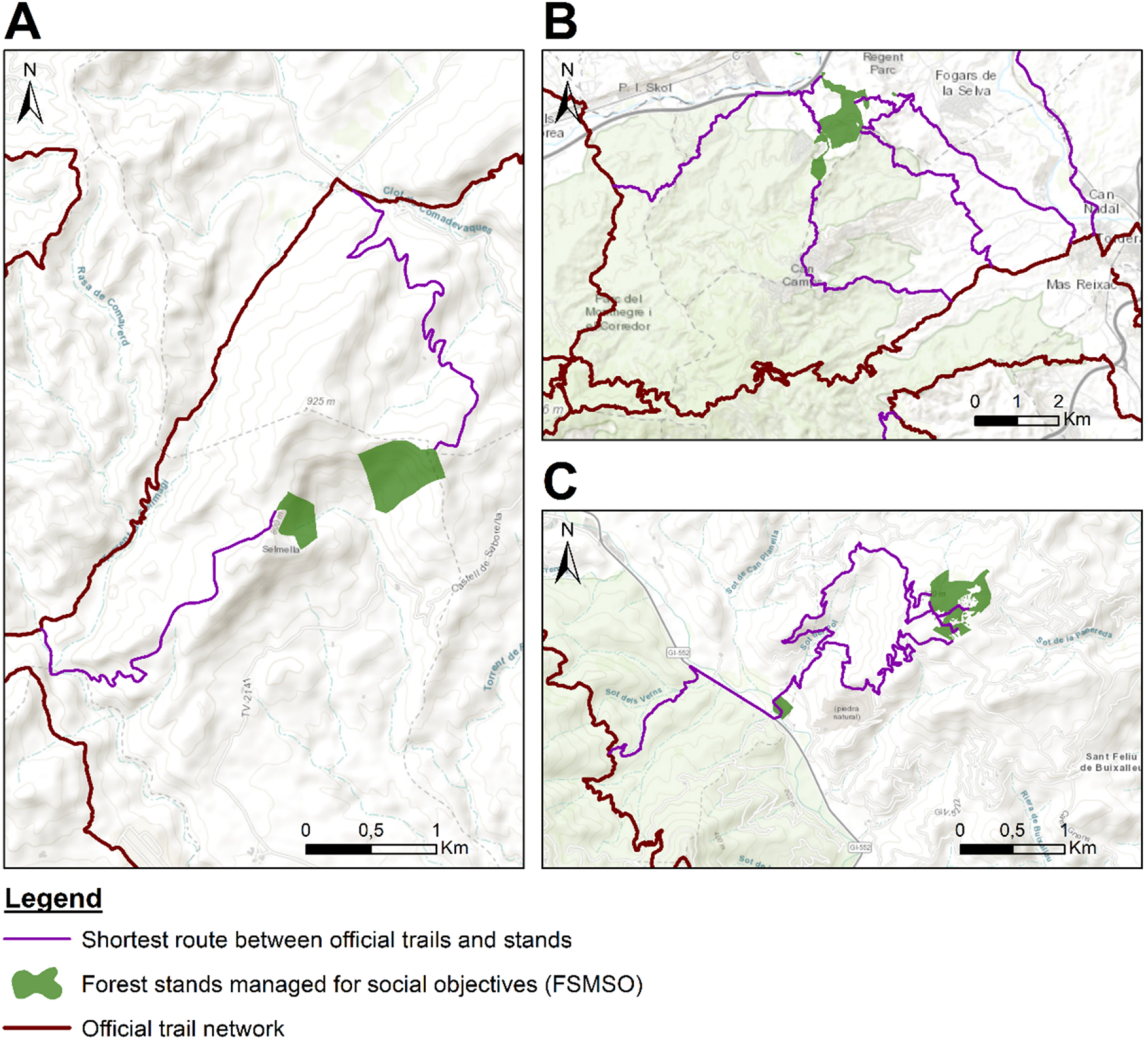
Examples of shortest routes that function as alternative routes of an official trail (**A**), multiple diversifying axes of the official trail network (**B**) and circular local trails (**C**). Note: The basemap is Esri Topographic, sourced from Esri, USGS, and NOAA. Accessed via ArcMap (2024)

Trail designers could thoroughly analyse our results to reveal additional purposes for the routes beyond simple round-trip connectors. For instance, sections could be homologated as alternative routes to official trails while providing access to the FSMSO (Fig. 10A). Diversifying the official trail network with multiple homologated routes (Fig. 10B) or promoting small and circular local trails that interconnect the stands with existing official trails (Fig. 10C) are also valuable alternatives to consider in an effective trail design process.

Nevertheless, as suggested by other authors (Courtenay and Lookingbill 2014), proposing routes and assessing their viability should include additional factors such as the physical characteristics of trails, logistical constraints and user preferences. In line with this user-oriented perspective, we acknowledge that additional variables (such as proximity to urban centres, the density of FSMSO, and the potential duration of recreational stays) are highly relevant and could enrich the viability analysis. These dimensions reflect spatial patterns of recreational demand and help to better align planning with users’ expectations and behaviours. We suggest that future research incorporate these aspects to strengthen the current approach and move towards a more comprehensive, user-centred evaluation framework. Including such factors would support managers in identifying and selecting the most suitable routes for connecting official trails with FSMSO in a more holistic and inclusive manner.

Achieving this requires detailed field planning and the active involvement of stakeholders. GIS and fieldwork are complementary in trail design (Àvila Callau et al. 2023) and both methodologies are crucial for effective trail planning and implementation. While GIS offers valuable planning tools and data, it cannot replace fieldwork decision-making in trail location (Snyder et al. 2008). Additionally, this study demonstrates that free, open-source GIS software is a practical and effective option for mapping forest accessibility (Sferlazza et al. 2021).

As a methodological limitation, it is significant to note that results may vary depending on the network layers used. We used the official road network, but it might miss some trails. A comparative analysis with various road networks would help identify discrepancies and evaluate alternative layers. Another concern is that some proposed connectors between official trails and FSMSO may already be officially recognised, such as tourist trails created by local authorities or mountain bike routes used by hikers. This limitation can be addressed in later trail design phases, where candidate trails undergo detailed evaluation and more comprehensive data collection.

Beyond its technical capabilities, the GIS-based methodology developed in this study serves a broader socio-humanitarian objective: enhancing access to forests for recreational and cultural uses. By identifying optimal connector routes between official trails and FSMSO, the approach supports not only spatial efficiency but also fosters social inclusion, well-being, and engagement with nature. These findings demonstrate that spatial analysis tools can be harnessed to operationalise social values, translating abstract concepts like CES into tangible, mappable outcomes. This bridge between spatial modelling and human-centred planning provides a replicable framework that forest managers and planners can adapt across varied contexts to improve recreational equity and the multifunctional use of forest landscapes. In this way, GIS does not merely assist in route location but also facilitates the intentional design of socially relevant, inclusive, and environmentally sustainable access strategies.

### Accessibility of FSMSO and the Role of Riparian Forests

FSMSO represented a small fraction of the study area, comprising just 3% of all forest stands, consistent with global patterns where only 6% of forests were designated for social or cultural services in 2020 (FAO 2020). Despite their scarcity, these stands showed moderate connectivity to the official trail network, with approximately 15% intersected by trails. Improving this connectivity is critical to enhancing access and maximising the CES they offer. Recreational trails play a crucial role in providing access to these stands, enhancing their appeal by connecting visitors to cultural or natural features of interest (Lukoseviciute et al. 2021). Therefore, developing methodologies like the one proposed in this study to link more FSMSO via recreational trails is vital for unlocking their full societal and environmental value.

In our accessibility analysis, approximately a quarter of the FSMSO were within a 20-min walk from official trails, half were more than 20 min away and the remaining could not connect to trails. Hippoliti (1976) categorised forest accessibility based on the time required for a round trip from the nearest road: areas accessible within 30 min were considered “served”, while those taking longer were deemed barely accessible or inaccessible. Considering this framework, the FSMSO in our study showed low accessibility from official trails. More specifically, individuals using the recreational trails may encounter difficulties in reaching these stands designated for social use, primarily because many are too far away or lack direct paths connecting the recreational trails to these specific areas. Such limitations could hinder the effective social use of these stands. Given that accessibility’s importance in viability scores was significant (40%), it is unsurprising that only 12.91% of routes achieved high viability in the viability assessment.

An analysis of FSMSO intersected by official trails by management sub-objective revealed that stands prioritising conservation (Preservation of forest spaces) were the most integrated into the official trail network. This trend was also evident in the created connector routes, where the highest percentage linked the official trail network to conservation-focused stands. These findings were unexpected, as conservation forests typically aim to minimise human impact to safeguard biodiversity and ecosystems. Increased accessibility in such areas risks unplanned tourism and heightened human pressure, potentially threatening their ecological integrity (Belonovskaya et al. 2019). Despite diverging from traditional norms, the growing emphasis on recreational trail development provides an opportunity to integrate recreation and conservation planning as showcased by several studies (e.g., Courtenay and Lookingbill 2014; Thomsen et al. 2014).

Regarding forest types, approximately one-third of the FSMSO consisted of *Quercus ilex* forests and riparian forests in equal measure. Across Catalonia’s planned forest stands, around 10% are *Quercus ilex* forests—one of the most abundant Mediterranean species (Ponce et al. 2022)-, while riparian forests account for only 3%. This high representation of riparian forests in FSMSO highlights forest managers’ recognition of their significant CES potential and growing social importance. Riparian forests are ecologically vital, contributing to water quality, flood regulation, nutrient recycling, and erosion control. They are also highly attractive for recreation and tourism (Saklaurs et al. 2022; Šrámek et al. 2018). Water bodies within forests enhance recreational value (Abildtrup et al. 2013), making riparian forests more attractive for recreation compared to other forest areas (Saklaurs et al. 2022). In Spain, water landscapes are increasingly preferred, especially by urban populations, leading to higher visitation rates in natural parks featuring riparian ecosystems (Vidal-Abarca Gutiérrez and Suárez Alonso 2013). Regarding CES tied to landscape aesthetics, studies consistently show a preference for structurally diverse forests, like riparian ones, over dense, single-species stands (Saklaurs et al. 2022).

Riparian forests were also among the most connected and accessible forest types in this study. Their linear configuration aligns with watercourses, naturally accommodating trails and promoting outdoor activities like hiking, trekking, and cycling (Virk et al. 2024). Consequently, it is expected to find riparian forests near the official trail network. This connectivity is particularly evident in riverscapes, where riparian trails serve as primary corridors for recreation (Grzyb 2024). Though less suitable for commercial forestry, riparian forests should be managed with an ecological focus while incorporating CES insights to balance ecosystem service synergies (Saklaurs et al. 2022). However, their high recreational appeal and limited spatial extent make them vulnerable to overcrowding. Effective management, particularly in high-traffic zones, is crucial to preserve their aesthetic, recreational, and ecological functions over the long term (Šrámek et al. 2018).

### Enhancing Recreational Access to Forests: Environmental and Property Challenges for Policymakers and Managers

The connector routes proposed in this study aim to expand the official trail network, enhancing access and social use of managed forests. This expansion offers recreational opportunities with physical and mental benefits but poses management challenges due to potential environmental impacts (Tudoran et al. 2022). As nature-based recreation grows in popularity, many natural areas face challenges of overcrowding and environmental stress (Dudney and Moghimehfar 2024). Recreational trails and their use can lead to habitat fragmentation, vegetation changes, surface erosion and altered animal behaviour (Tomczyk 2011). The severity of these effects depends on factors like activity type and intensity, participant numbers, species or habitat type sensitivity and cumulative impacts from other land uses and recreational activities (Pröbstl 2003). It is essential to recognise that new trails can attract more visitors, potentially causing overcrowding, natural resource degradation and other environmental impacts (Tudoran et al. 2022), both around trails and FSMSO.

Identifying and managing the “recreation carrying capacity”—the maximum number of visitors an area can support without environmental damage—is crucial (Dudek 2017). In relation to this concept, a “spiral of improvement” may occur, where new infrastructure attracts more visitors, necessitating further development and exacerbating overcrowding (Ciesielski and Stereńczak 2018). Another significant impact derived from trails and increased access to natural environments is wildfire risk. In the Mediterranean region, especially during summer, human presence in natural spaces—whether from visitors, residents, or workers—poses a significant risk for forest fires (Boada et al. 2005). Consequently, seasonal access restrictions are often imposed to mitigate these risks (Brown and Jenkins 2023). In our study area, in recent years we have seen the implementation of accessibility restrictions in protected natural spaces during summer wildfire risk emergencies.

This study connects forest stands using existing road networks, reducing habitat fragmentation but raising issues like route ownership. The results showed that approximately 70% of the total length of the created routes passes through trails and paths with unknown property. Many of these paths may be private and run through private properties. Furthermore, the stands managed for social use in this study are part of technical forest management plans primarily promoted by private landowners. This situation can lead to potential conflicts between private landowners and trail users or FSMSO users.

Although the ownership of many of these routes is unknown, which may result in access restrictions, our analysis prioritised minimising ecological disruption by leveraging the existing trail network. We acknowledge that the lack of information regarding road management authorities presents a challenge for the effective implementation and governance of proposed connections. Addressing these potential conflicts is therefore crucial for the homologation process of new routes (Corning et al. 2012). Engaging local communities, landowners, and relevant stakeholders in planning and decision-making processes can help mitigate these issues and enhance both the legal certainty and social acceptance of future trail connections (Courtenay and Lookingbill 2014; Wiggs et al. 2008).

Landowners play a pivotal role in granting public access but often cite concerns about privacy, trespassing and irresponsible trail use, including issues like parking on private property, unleashed dogs and property damage (Moore 1994). However, these concerns can vary significantly depending on factors such as the territorial context, making the issue complex and multifaceted. For instance, in Bergstén et al. (2018), many private forest owners in Sweden expressed acceptance of public roaming on their lands, reflecting the importance of the Right of Public Access to nature in Sweden. Unlike in our study area, many European countries like Sweden have regulatory frameworks granting public access to wildlands for activities such as hiking and camping, regardless of ownership (Hammitt et al. 1992). This tradition supports balancing property rights with public access, fostering social responsibility. However, private landowners recognise the costs associated with public access, which can conflict with their management goals (Daigle et al. 2012). Studies, including Daigle et al. (2012) and Johnsson and Beery (2022), show that landowners with strong ties to their land tend to limit recreational activities, especially those incompatible with their priorities.

Studies regarding the potential conflict between recreational trails and private forest owners have yet to be identified in our study area. However, conflicts related to specific cultural activities, such as mushroom picking, exemplify this issue. Górriz-Mifsud et al. (2017) surveyed private forest owners in Catalonia and found that many perceived mushroom pickers as a nuisance due to congestion. Consequently, many forest owners supported regulations to enforce good picking practices. To mitigate conflicts, policymakers should create legal frameworks clarifying rights, responsibilities, and enforcement mechanisms while incorporating landowners’ perspectives. Integrating private ownership dynamics into planning can enhance public access and align with management goals (Bergstén et al. 2018; Górriz-Mifsud et al. 2017).

### Management Recommendations

Designing new trails and managing existing ones requires addressing multiple considerations, with ecological factors being particularly critical (Tomczyk 2011). Drawing from our findings and the discussions presented in the previous sections, we propose the following recommendations as practical guidelines to promote sustainable recreation while preserving ecological integrity. These suggestions are intended to mitigate the socio-recreational impacts associated with expanding access to forest stands, especially those designated for social use:

- Environmental impact assessment: Conduct thorough impact assessments before homologating new routes.
- User management: Implement strategies such as recreation carrying capacity evaluations, prohibiting high-impact activities, and providing user education to prevent overcrowding and manage flow effectively. Promoting a diverse regional network of historical, cultural, and recreational attractions can help disperse visitors, reducing pressure on overcrowded areas (Brown and Jenkins 2023). In this line, the connecting routes proposed in this study offer a valuable tool for redistributing visitor flow to areas with lower biodiversity impacts or wildfire risks.
- Maintenance and monitoring: Regularly maintain new and existing trails to prevent degradation and monitor environmental impacts continuously.
- Stakeholder engagement: Involve local communities and stakeholders, namely forest owners, in the planning and decision-making to ensure the trails meet user and owner needs.

## Conclusions

This study introduces a new method to optimise forest use for socio-recreational purposes. This method strengthens the connection between forest planning and trail design while offering scalability for application in other regions. Using GIS, the approach identifies routes connecting official recreational trails to forest stands managed for social objectives, evaluating both stand accessibility and the viability of proposed routes. This allows decision-makers to prioritise the most viable routes early in the planning process, streamlining their consideration for future homologation. Additionally, the analysis offers flexibility to explore options beyond standard round-trip connector routes. The findings highlight limited real connectivity between official trails and forest stands managed for social objectives, as well as low potential accessibility via the shortest generated routes. Given the growing demand for CES, forest managers and trail designers can utilise these insights to improve socio-recreational opportunities. However, these efforts must balance accessibility with environmental protection and respect for private landownership. A noteworthy outcome is that riparian forests are strongly represented among the forest stands managed for social objectives and emerge as the most connected type of forest. While primarily valued for their regulatory and ecological roles, this study underscores their significant socio-recreational potential, advocating for their integration into broader forest planning frameworks.

## Data Availability

No datasets were generated or analysed during the current study.
